# Convergence of Precision Medicine and Public Health Into Precision Public Health: Toward a Big Data Perspective

**DOI:** 10.3389/fpubh.2021.561873

**Published:** 2021-04-06

**Authors:** Pedro Elkind Velmovitsky, Tatiana Bevilacqua, Paulo Alencar, Donald Cowan, Plinio Pelegrini Morita

**Affiliations:** ^1^School of Public Health and Health Systems, University of Waterloo, Waterloo, ON, Canada; ^2^David R. Cheriton School of Computer Science, University of Waterloo, Waterloo, ON, Canada; ^3^Waterloo Artificial Intelligence Institute (Waterloo.ai), Waterloo, ON, Canada; ^4^Institute of Health Policy, Management, and Evaluation, Dalla Lana School of Public Health, University of Toronto, Toronto, ON, Canada; ^5^eHealth Innovation, Techna Institute, University Health Network, Toronto, ON, Canada

**Keywords:** precision medicine, public health, big data, data analytics, artificial intelligence, precision public health, systematic review

## Abstract

The field of precision medicine explores disease treatments by looking at genetic, socio-environmental, and clinical factors, thus trying to provide a holistic view of a person's health. Public health, on the other hand, is focused on improving the health of populations through preventive strategies and timely interventions. With recent advances in technology, we are able to collect, analyze and store for the first-time large volumes of real-time, diverse and continuous health data. Typically, the field of precision medicine deals with a huge amount of data from few individuals; public health, on the other hand, deals with limited data from a population. With the coming of Big Data, the fields of precision medicine and public health are converging into precision public health, the study of biological and genetic factors supported by large amounts of population data. In this paper, we explore through a comprehensive review the data types and use cases found in precision medicine and public health. We also discuss how these data types and use cases can converge toward precision public health, as well as challenges and opportunities provided by research and analyses of health data.

## Introduction

Over the past decade, there has been an increased interest in the field of precision medicine. This field explores the development of targeted treatments for individuals based on genetic, environmental, clinical, and social factors. The National Institute of Health defines precision medicine as an “approach for disease treatment and prevention that takes into account individual variability in genes, environment, and lifestyle for each person” (1), with the goal of accurately identifying which treatments and preventions will be more effective in which population groups (2). Precision medicine is about having a holistic understanding of an individual's health to create more precise treatments or prevention programs for specific traits and profiles in a population (3). For example, genetic information from patients with a shared disorder can be used to develop new drugs that could be used in the population with this shared disorder (3, 4).

The Center for Disease Control (CDC) defines public health as the “science of protecting and improving the health of people and their communities” (5). While in precision medicine the unit of interest is the individual, public health views populations as the basic unit for interventions. This is achieved through prevention practices and interventions in a population. From the previous definition of precision medicine, we can extend the concept to include “precision public health”: the study of interactions between biological and genetic factors with personal, environmental and social determinants of health, to monitor the incidence of diseases in communities and target effective interventions in the population (3). This would involve, for example, stratifying populations according to specific traits, behaviors or genetic information, to achieve better intervention and treatment outcomes.

Frontiers in Public Health defines precision public health as “the application and combination of new and existing technologies, which more precisely describe and analyse individuals and their environment over the life course, to tailor preventive interventions for at-risk groups and improve the overall health of the population” (6). More succinctly, Khoury described precision public health as “the ability to prevent disease, promote health and reduce health disparities in populations” with the use of emerging methods and technologies (7). These emerging methods involve the application of techniques for analyzing large quantities of diverse data, enabled by several advances in health data collection. Among these advances we can cite smart technologies collecting patient-generated health data (PGHD), Electronic Health Records (EHR), and genomic sequencing.

Regarding the first topic, society is currently moving into an age of ubiquitous and smart technologies that continuously monitor our health, enabling the collection of PGHD such as physical activity levels, heart rate, and blood pressure. These can be combined with different data types, such as social data from several social networks, to provide a more complete and hopefully accurate view of a person's lifestyle and health behaviors (8–10). The potential for smart technologies to provide new, rich, and diverse health data is immense because of their popularity: a 2016 report showed that 3 out of 4 Canadians owned a smartphone (11), for example. Even in developing countries, smartphones are a reality: in Brazil, for instance, 57% of the population has one (12). The wearable market has also experienced rapid growth: the number of global smartwatch users increased from 5 million in 2014 to 141 million in 2018 (13). Moreover, wearable owners are represented in all age ranges, not just in young people (14).

Additionally, advances in EHR systems have greatly advanced healthcare and are currently widely in use (15). In the U.S., for example, the Department of Health and Human Services Meaningful Incentive Programs encouraged 95% of hospitals to adopt Electronic Medical Records or EHRs in the past 10 years (16).

Finally, the genomics field is also experiencing several changes. The sequencing of the first human genome happened in the early 2000s after decades of work and a cost of approximately USD 3 billion. Today, next-generation sequencing technologies that read DNA molecules in parallel, make it possible for individuals to get their genomes sequenced for about USD 1,000 (17–19).

All these technological advances have led to an explosion of high volume, high velocity, and high variety health data. These so-called 3 Vs are what define datasets as Big Data (8–10, 20, 21). Since its introduction, many authors have expanded the definition of Big Data to include many more “Vs.” For example, in a systematic literature review of methods and challenges for Big Data, Sivarajah et al. (22) report finding discussions with 4 Vs (adding variability), 6 Vs (adding veracity and value), and 7 Vs (adding visualization). It is important to differentiate here the term Big Data and associated technologies, which refers to the “Vs” and the technologies needed to process, store and analyze these data; and the term Artificial Intelligence (AI), which in this paper will refer to the methods and algorithms used in creating advanced computer programs capable of learning and adapting through several iterations of data to achieve a prediction or outcome.

Big Data technologies and techniques can help researchers make sense of the plethora of rich and diverse health data currently being generated. Data analysis is essential, as society has reached a point in which data generation surpasses our capacity to gain knowledge from the data without computational support (8). In order to perform these analyses, the use of powerful computing and storage technologies, allied with AI algorithms to make sense of and gain insight about Big Data, is needed.

**Figure 1:**
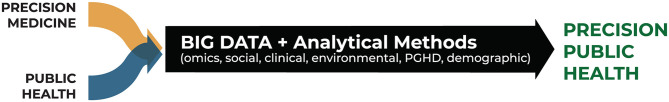
Big Data is the glue that brings precision medicine and public health together, allowing researchers to study interactions between comics, clinical, social, and environmental data.

Advances in computing power, data gathering technologies and storage supplies the resources for researchers to study the interaction on large datasets, including genomics, environment, and social networks, as well as other types of health and personal data. If precision medicine and public health are going to use the new healthcare data being generated by emerging technologies, Big Data is the field of computer science that supports the use of these data, identifying patterns and insights. To make these analyses possible, AI and statistical analyses methods are applied. This relationship is illustrated in Figure 1.

**Figure 2:**
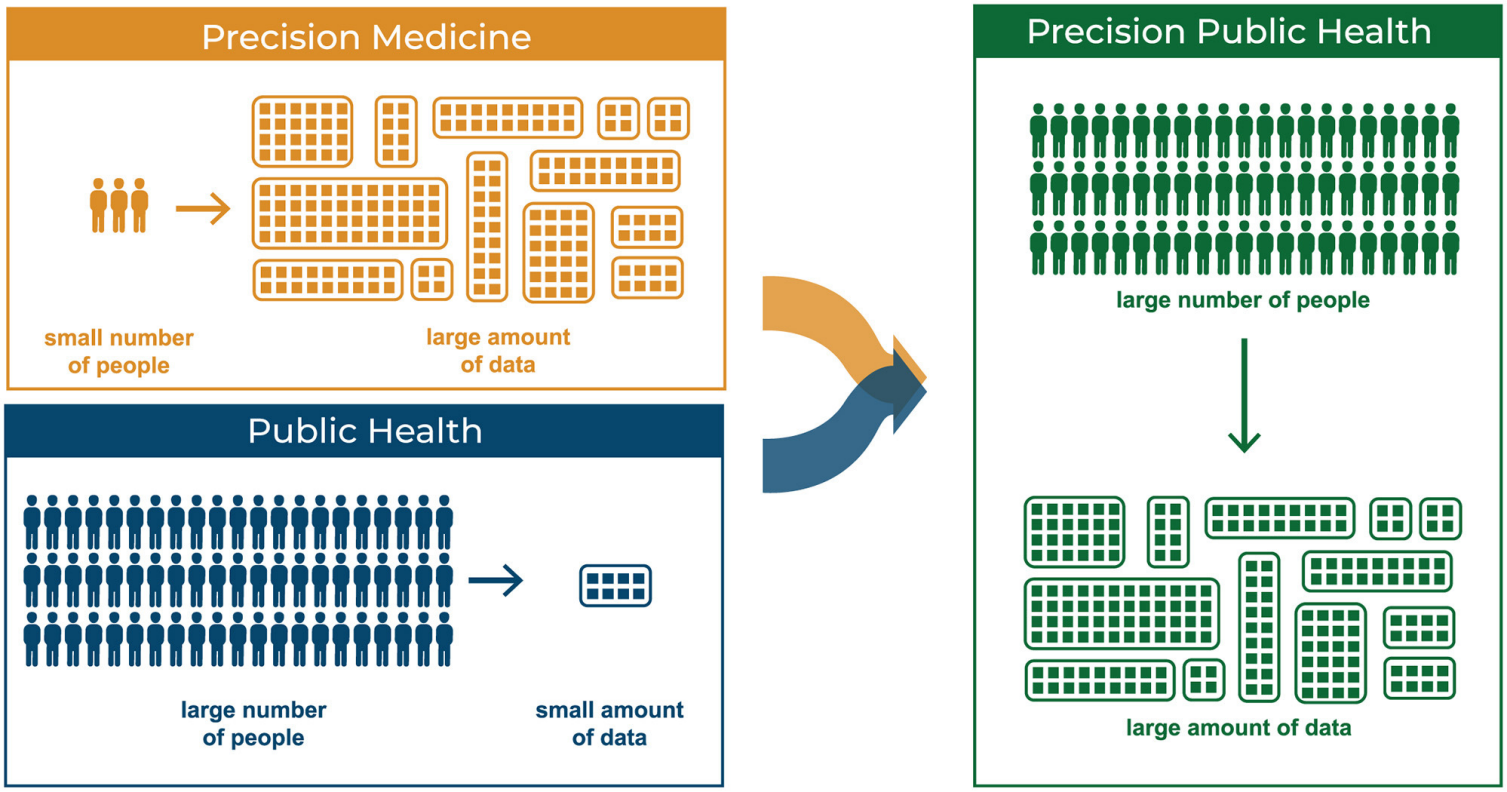
Precision Medicine: few people providing a large number of types of data. Public Health: large number of people providing limited types of data.

In addition, in contemporary healthcare, precision medicine and public health are mostly seen as different fields of research. This is despite the fact that health data used by both fields is, for the most part, the same. Both fields require a huge amount of data: in precision medicine researchers usually collect many different types of data from few people, whereas in public health, they gather a few data types from many people. In truth, more data collected from as many people as possible should yield better results from statistical analyses. For example, only with a large enough cohort can genomic variance be detected (23). We argue that precision medicine and public health are complementary, as shown in Figure 2; further, if Big Data is the glue that holds all this health data and analytics together for precision medicine and public health, it can also help to promote a convergence between the two fields into precision public health.

Dolley (9) defines four main areas in which Big Data can improve precision in public health: Disease Surveillance and Signal Detection, Risk Prediction, Targeting Treatment Interventions, and Study of Disease. In this paper, we will explore how the recent Big Data explosion can help toward the convergence of precision medicine and public health into precision public health. We will review what health data is important for public health and/or precision medicine. Then, we will discuss how several of the identified data uses can lead to precision public health, using Dolley's main areas to frame the discussion.

Our goal is to (i) provide an overview of which Big Data types and usage are relevant to precision medicine and public health, through a literature review; (ii) to discuss opportunities and challenges concerning the convergence of precision medicine and public health into precision public health. While there have been many publications discussing the emergence of precision public health and its merits, we intend to describe the data types and its uses for precision medicine and public health systematically, in order to discuss the importance of the novel field of precision public health.

The rest of the paper is divided as follows: the Methods section describes the methods that we used to conduct the literature review. The Results section provides an overview of the main data types, sources, and uses, and presents how combinations of these data can be used for precision medicine and public health. The Discussion section discusses how precision medicine and public health can be brought closer together with a data-driven approach, and the challenges related to this approach with a special focus on privacy issues. The Conclusion section presents concluding remarks, limitations, and future work.

## Methods

To achieve our goal, we conducted a review. The team followed a framework (24) to make sure the findings were analyzed systematically. The framework consists of the following six stages: (i) identifying the research question; (ii) identifying relevant studies; (iii) study selection; (iv) charting the data; (v) collating, summarizing and reporting results; and (vi) consultation, which is optional.

### Identifying the Research Question

To achieve the two goals defined in the introduction, the paper will answer the following research questions: (i) What are the relevant data types related to precision medicine? (ii) What are the relevant data types related to public health? (iii) What are the relevant uses of data in precision medicine? (iv) What are the relevant purposes of data usage in public health? (v) How can the uses of data types raised in the previous questions promote convergence between precision medicine and public health into precision public health?

### Identifying Relevant Studies

The review was conducted on the following health and informatics databases: IEEE, Google Scholar, PubMed, and Web of Science. We used the following strings related to general terms of the field: “Precision Medicine,” “Personalized Care,” “Precision Health,” “Public Health,” “Population Surveillance,” and “Precision Public Health.” Each of these terms were searched in combination with the following keywords/search strings, pertaining to methods of data processing or analytics: “Big Data,” “Artificial Intelligence,” “Machine Learning,” “Deep Learning,” “Regression,” “Clustering,” “Analytics.”

As stated by Khoury (3), the terms “precision medicine” and “personalized medicine” have often used interchangeably, although there is an increasing preference for the former. Originally we had included the term “personalized medicine” in our review for completeness. However, as we proceeded with the review we realized only one of our references included in our article included the term “personalized medicine.” On a PubVenn search of the two terms, only about 9% of articles for precision medicine and personalized medicine overlap at this time (25); therefore, we decided to remove this term from our review. For more details, the difference between the terms is described elsewhere (3, 4, 26). We also included some of the more common techniques associated with statistical methods, mainly related to Artificial Intelligence (AI).

### Study Selection

The main exclusion criteria were papers that did not deal with Big Data or associated technologies and methods. To this end, the team used the definition of the term Big Data rather than checking whether the term was present. Additional restrictions include practical concerns of availability and language (only English references were included, except for data about mobile device usage obtained from a Brazilian website).

### Charting the Data

This stage consists of defining which information will be extracted and analyzed from the results of the review. We focused on the following information:

- What data types are being cited/used?
- What is the purpose of the usage of data?
- Is the focus on precision medicine, public health, precision public health, or a combination of these fields?

### Collating, Summarizing, and Reporting the Results

For each result, we mapped what data types were cited and their purpose, as well as the fields in which the data was collected. Concerning research questions (i) and (ii) in our literature review, we found four major data types that are used by precision medicine and public health: omics, clinical, social, and environmental. In this subsection, we describe each of these data types and their sources (meaning how and where they are generated or collected). As we describe the different data types we found in the literature, we provide examples regarding their current use, whether in precision medicine or public health—although certain data type uses are often associated with both. We go into more detail on these uses in the following sections, exploring how each of these data types can be combined for both precision medicine and public health. Table 1 shows a summary of our results.

**Table 1:** Data Types Identified in the Literature Review.

**Data Types**	**Omics**	**Clinical**	**Social**	**PGHD**	**Environmental**	**Demographic**
Definition	The study that explores the roles, relationships, and actions of the various types of molecules	Data collected through the course of treatment or in the processes of clinical trials	Information publicly shared on social media and related to personal health data collected by the individual	Information on the health and behavior of individuals collected through personal smart devices	Information gathered from the context in which individuals and populations are immersed	Information describing attributes of the population under study
Examples	Genomics, epigenomics, proteonomics, transcriptomics, etc.	EHR, laboratory tests, MRI, CT Scans, Administrative data, etc.	Social media posts, GPS location, data generated through smartwatches, smartbands, etc.	Smart personal devices data such as sleep, heart rate, and physical activity	Natural resources quality, temperature, crime rates, traffic, walkability of neighborhoods, etc.	Age, sex, education, income, ethnicity, employment, etc.
Possible Uses	Oncology and genetics studies, pharmacogenomics, omic biomarkers, clinical trials improvement	Predictive medicine, trends and correlation identification, clinical trials improvement, false alarm mitigation	Social media use, behavior and social habits assessment (e.g., quit smoking, lose weight, etc.)	Health self-management and research into behavioral aspects of an individual (e.g., dietary intake tracking, vital signs log, etc.)	Air and water quality monitoring, traffic impact measuring, social factors impact on life quality	Stratifying populations under study in groups with the same attributes, preventing biases and confounding, and serving as a normalization tool for comparing data points in a study

## Results

### Data Types, Sources, and Uses

This section focused on defining data types being used by precision medicine and by public health. We provide a brief definition of each data type, discuss possible sources for data collection and examples of how these data can be used. The next section provides more details into the use and combination of these data in precision medicine and public health.

#### Omics Data

##### Definition

Omics (or -Omics) studies the features of molecule profiles (e.g., genes, proteins, metabolites) to understand the relationship between molecules of an organism better (27, 28). Data commonly studied include genes, chemical compounds, proteins, metabolites and carbohydrates, among others (27, 29, 30). The study of biological factors and their interactions, including the relationships between the several “-omics” types, are particularly essential in the field of precision medicine. These studies can lead to several insights, such as understanding if there are different subtypes of a disease and studying factors that might make a potential drug more effective (31).

##### Sources

These data are mostly obtained from sources such as next-generation sequencing technologies (NGS) (28, 29, 32, 33). NGS technologies analyze multiple chains of DNA in parallel, speeding up the process (indeed, an entire human genome can now be sequenced within a day) and making it more cost-effective. An example of NGS technologies is deep sequencing, which involves the sequencing of the same gene a multitude of times to detect rare mutations or variations in a cell (33, 34).

##### Examples of Uses

A possible use for omics data include studying genetic traits to tailor treatments. For example, many studies can relate diseases such as obesity and cystic fibrosis to genetic factors (35, 36). Even communicable diseases, such as influenza, can have genetic factors that have implications in an individual's resistance to treatment (23).

Another use of omics data is the detection of biomarkers, which can help researchers to better understand certain diseases. Biomarker detection can also be of great importance in improving clinical trials. A tool to track biomarkers can allow the measurement of differences in patients at a biological level, allowing researchers to assess the effects of a drug much faster and more accurately than current methods permit (31). Omics data and genomic biomarkers, associated with computational agents like Machine Learning algorithms, have the power to discover novel associations between molecular profiles and other clinical variables that would not be assessed otherwise and can lead to the identification of new clinically-relevant subtypes in clinical trials (37, 38).

#### Clinical Data

##### Definition

Clinical data is collected from patients during treatments or in clinical trials, usually at a medical facility. Examples of clinical data include laboratory tests, such as CT Scans, ECGs X-Rays. Some subtypes of clinical data can be collected and managed solely by individuals using health devices such as wireless scales, smartwatches, and blood pressure monitors. However, these sources and uses of clinical data are not officially monitored in medical facilities and registered on patients' EHRs. Therefore, in this paper, we are considering clinical data as all data that can be obtained and stored within healthcare facilities and by providers, and that definition is used in the points below. For data generated by health devices, see next subsection on social data.

##### Sources

Clinical data is typically collected from the following six sources: Electronic Health Records (EHR), Administrative Data, Claims data, Patient/Disease registries, Health Surveys, and Clinical Trials Data (39).

##### Example of Uses

Clinical data can be used to predict individuals' diagnoses, reaction to treatments and survival outcomes (8, 31, 40, 41). There have been several research programs using Deep Learning for this purpose. For example, a researcher at Trinity College trained a Machine Learning model on 110 MRI scans that achieved a 90% accuracy in early diagnosis of Amyotrophic Lateral Sclerosis (31). Gulshan et al. (42) developed Deep Learning algorithms that exceeded the performance of specialists in detecting diabetic retinopathy and diabetic macular edema in retinal fundus images.

Clinical data can also be coupled with remote monitoring technologies to monitor patients within and outside hospitals without the need of physical contact—which is essential during the current COVID-19 pandemic. For example, Dhillon et al. (43) proposes a system that processes events from wearable sensors collecting ECG, EEG and Blood Pressure data. This system also has the ability of providing alarms in case of an emergency event.

#### Social Data

##### Definition

A straightforward definition of social data is information shared publicly on social media, including information on the user's location, circle of friends, and language (44). Expanding this concept, the usage and meaning of certain data are closely associated with social interactions (such as behavioral data, even though they were not generated nor collected through social media).

##### Sources

These include social media such as Facebook, Twitter, and Google (e.g., search history, Google Trends), and data representing social interactions.

##### Examples of Uses

Social data can be used to estimate behavioral characteristics of an individual. For example, 20% of patients with chronic conditions share their experiences online with other patients, creating communities on social media (10). These data can be leveraged for health purposes. Food images from Instagram can be used to study dietary behavior of adolescents. Deiner et al. (45) used Twitter data to identify the occurrence of diagnosed conjunctivitis. In addition, more advanced studies are capable of marker identification and even prediction of diagnosis: Reece and Danforth (46), for example, used Machine Learning to identify markers of depression using Instagram photos, Jain et al. (47) use Twitter data to predict insomnia, and Odlum et al. (48) showed that an increase in Twitter data about Ebola indicated increased incidence in Nigeria three days before the news alert and seven days before Centers for Disease Control official alerts. However, one has to be careful about using social data as many of these research results may not be repeatable. In addition, researchers need to be mindful of users' privacy as these data may not have been originally collected as part of a research study (as expanded in the Discussion section).

#### PGHD

##### Definition

PGHD, or “self-generated,” real-world data, includes information on the health and behavior of individuals collected through personal smart devices. Many data types in the literature related to personal clinical data, such as weight and heart rate, are not officially considered for clinical use by caregivers but can be used by individuals to self-monitor their health and behavioral goals (e.g., quit smoking, drink water, exercise, etc.) and are included in this definition (20, 49–53).

##### Sources

Data collected through personal mobile/wearable devices such as smartphones, smartwatches, smart thermostats, among others.

##### Examples of Uses

Applications such as Apple Health and Google Fit can collect and manage individual data such as exercise routine, calorie intake, heart rate, and weight, among others (49, 50, 52, 53). There is mixed evidence on the accuracy of data collected through smart sensors, and many factors can affect it (54, 55). A recent systematic review of wearables found that data may be under- or overestimated in several devices and models (55), and a study comparing FitBit Flex and the ActiGraph GT3X+ found that the Fitbit significantly underreported steps in free-living conditions (56).

In addition to health self-management, smart devices can also help researchers collect data for health studies. For example, ResearchKit is a framework developed by Apple to help researchers create apps that recruit study participants and collect health data from connected devices (50). An example is mPower, an iOS app that measures balance, dexterity, and gait with iPhones' accelerometer and gyroscope to understand Parkinson's disease better. It has more than 10.000 users (93% never took part in any study) and became the largest Parkinson's study in history (50, 51). Of course, there need to be many more studies on whether the accuracy of this data is adequate to support research.

#### Environmental Data

##### Definition

Environmental data is related to information gathered from the context in which individuals and populations are immersed. Examples of environmental data include air pollution, temperature changes, water quality, crime rates, and walkability of a neighborhood, among others.

##### Sources

These include air pollution and weather sensors, GPS, GIS, Police Data, Public Transportation Databases, and mobile apps.

##### Example of Uses

It is well-known that certain environmental factors can directly affect our health, such as air and water quality. Natural and man-made impacts on natural resources are related to several acute and chronic diseases. These impacts can be assessed to develop better healthcare strategies for individuals and populations, although the latter is more often associated with these concerns. Off-the-shelf sensors such as Ecobee smart thermostats, for instance, are new solutions that collect several environmental data variables (57–59).

Other factors related to the built environment, such as incidence of crime, transportation, and city planning, can have a significant impact on individual and population health. Many reports show that factors such as commuting may have a negative impact on health, affecting our metabolism (e.g., raising blood sugar and cholesterol levels), posture, and interfering with sleep patterns (60).

#### Demographic Data

##### Definition

This includes any data related to the description of individuals or populations under study, such as age, sex, ethnicity, education, employment, and income (61).

##### Sources

There are many ways to obtain demographic data. Participants can self-report their demographic characteristics when a study begins, for example. Researchers can also access data from large surveys and census which typically collect this information, such as the Canadian Health Measures Survey (62) (further described in the Discussion). This information can also come in clinical records of individuals (in this manner, they can be seen as a form of Clinical Data) and also be collected through social media (in the same manner, data collected in this way would also be classified as Social Data for our purposes).

##### Example of Uses

Demographic data are typically used to stratify participants in a study according to certain characteristics, for example to identify if a certain factor is present or not in the sample of participants with the same characteristic (61, 63, 64). The use of demographic data can also lead researchers to identify and avoid any biases or confounding, as well as to examine the validity of the study as analyses performed in a certain population may not be directly translated to populations with different characteristics (64). In this manner, demographic information acts as a normalization tool to ensure that the collected data of individuals can be compared pertaining to the variable or outcome of interest on the same basis, minimizing the probability that no other factors may cause incorrect analysis or results (63).

### Data Interactions in Precision Medicine and Public Health

In this section, we address research questions (iii) and (iv) by describing the use of the four data types defined in the previous section for precision medicine and public health. We outline current uses of these data types, as described in the literature, and include possible combinations of the aforementioned data types. The uses proposed here implicitly involve demographic data as this information is typically used for analyses in studies as described in the previous section. There are many results that can be inferred by combining data types, thus expanding the scope of both these fields.

#### Precision Medicine

##### Omics

As previously mentioned, Omics data are extensively related to precision medicine, as they can help tailor treatments and interventions specifically to an individual's profile. The first advances in precision medicine were mostly driven by the field of oncology since it is long known that cancers are essentially genetic abnormalities. By analyzing omics data, it is possible to identify the molecular profile of the tumors, leading researchers and clinicians to understand better their specific mutations (36, 38, 41, 65). In addition, many studies are now assessing omics data to relate phenotypical outcomes with genetic markers better. For example, Chen et al. (66) demonstrated the critical role of the GRIN3A gene in nicotine dependence, a key factor that may affect the success rate of people who try to quit smoking. The use of omics data for biomarker detection can also further be assessed for identifying new population stratifications that target identification and comparative effectiveness (40).

##### Clinical Data

An interesting application of clinical data in precision medicine is to improve clinical trials. Models that accurately predict diagnoses can lead to better treatments for patients, which in turn will lead to better outcomes as well as a better understanding of the underlying causes of diseases. Diagnostic prediction can also minimize the risk of misclassification of patients in trials, making their results more accurate. In addition, companies like Origen Data Sciences want to use Machine Learning to create “virtual patients” for the trials. In short, instead of the traditional clinical trial model in which patients are divided into groups, with a comparison group that will receive a placebo drug, the goal with “virtual patients” is that all patients would receive drugs and be compared to a computer model of how they would have progressed in the treatment if they had been placed in the control group (31, 67).

##### Omics and Clinical Data

Considering omics and clinical data, Johnson et al. (40) describe how medication dosing is a huge problem in healthcare, particularly in ICUs. Studies conducted in institutions show that, because of complex factors involving dose-response relationships, it is very hard for medical staff to assess the appropriate dosage for each patient. To that end, there have been several research programs that try to estimate medication dosage through analytic techniques, such as linear regressions (68) and reinforcement learning (40, 69), applied to omics and clinical data. Clinical-genomic data can also be considered closer to real-world data, useful not only for delivering care but also to support the design of future clinical trials since it could provide better site selection and patient recruitment criteria (41).

##### Clinical, Social and PGHD Data

From a precision medicine perspective, there is little, if any, division between clinical, social or PGHD data. The source of the data is not as important as having accurate information that reflects a patient's history. However, knowing the source of the data can help researchers to understand its accuracy better; typically, data from clinical sources are highly accurate, but represent few measurements over a short period of time. Social data and particularly PGHD, on the other hand, represent a large volume of data that may help in identifying outliers and filter variations, but may also contain more gaps and innaccuracies. Since the purpose of precision medicine is to deliver more precise care, individual health data is of the utmost importance. The popularity and ubiquity of smart technologies, coupled with increasingly advanced methods and services of storage and analytics platforms (21), allows for the creation of real-time feedback loops for the patients (10, 20). For example, an individual that uses smart devices such as smart bands, smartwatches or smartphones can have their health data collected continuously and effortlessly. This data can be further analyzed and the results of these analyses can be provided as feedback given to the user in near real-time. These results can tell the user, for example, if he exercised enough during the day or if he needs to exercise more to compensate for not doing enough physical activity in the previous week. In this case, the analyses of the data “bypasses” the need for a doctor and very quickly provides feedback on individual health, as well as treatment or prevention strategies tailored to the individual. It is important to note that health data collection, particularly PGHD, make it essential for data collectors to handle the data in an ethical way and lead to a discussion about data accuracy, ownership, privacy, and consent. Johnson et al. (40) indicate that privacy is one of the main factors that can impact health data collection and use; this is an important consideration for all data types mentioned here in the article, but from a research and analytics standpoint PGHD may provide additional difficulties as this information typically is not collected primarily for research purposes. We provide a more detailed discussion of privacy in section Security and Privacy of Personally Identifiable Information (PII).

##### Social, PGHD, and Environmental Data

Although clinical data is the more obvious data type in which analytics can be run and insights into individuals health and behavior generated, Barret et al. (20) proposes that socio-environmental data can also be integrated for more analyses and to encourage healthy lifestyles. For example, social networks could be used to link groups with common interests and who live in the same location to increase physical activity (52, 59, 70–72). Hicks et al. (63) describes examples of studies using social and environmental data, for example by linking smartphone-based physical activity with city walkability data to analyze activity inequality.

#### Public Health

##### Clinical Data

Chimmula and Zhang (73) used a relatively small Canadian dataset on fatalities and recovered patients to model the virus' transmission, achieving 93.4% accuracy for short term predictions (although the researchers found that the pandemic would continue in Canada until June of 2020, which was not the case, suggesting that long-term predictions are not valid; a large dataset can potentially represent more accurate information).

##### Omics and Clinical Data

Prevention is a major public health goal. An essential step for prevention is identifying risk factors for diseases, which range from genomic factors to physical activity, tobacco use, and pollution, among others. Advanced analytical techniques can help in identifying risk factors and correlations among variables (20). An example includes state run public health programs in the U.S. which screen more than 4 million newborns yearly to detect genetic or metabolic conditions (74).

##### Clinical and Social Data

Google search data has been used to identify suicidal ideation, while Twitter data can be used to assess insomnia at a population level (47). Another example of multi-data usage for public health is targeting effective interventions to specific subgroups in the population, as proposed by Barret et al. (20).

During the COVID-19 pandemics, we have seen several papers which use advanced analytics to try and predict the spread of COVID-19 in a certain region. For example, Ayyoubzadeh et al. (75) analyzed a dataset comprised of daily incidence of COVID-19 in Iran and Google Trends data, specifically looking for search terms appearing in queries like “corona,” “antiseptic selling” and “hand sanitizer” (as an example of integration of clinical and social data). While accuracy was not reported, the model's Root Mean Square Error was 27.187. The authors found evidence of overfitting in the model because of the limited amount of training data and suggested that more data will lead to better accuracy, again suggesting the potential of advanced analytics combined with large volumes of data. Qin et al. (76) were able to predict COVID-19 cases successfully using search indexes from Baidu's, China's more popular search engines. Arguably, these cases could be branded precision public health owing to their use of Big Data and a more precise understanding on the behavior of individuals; we decided to include them here as they target entire populations as opposed to specific groups or regions and therefore may not represent a truly precision approach.

##### Clinical, Social, and Environmental Data

Ram et al. (77) used social media data (Twitter data and Google search queries) coupled with environmental data (air-quality data) to predict clinical data (daily emergency visits for acute asthma) accurately.

##### Omics, Clinical, Social, PGHD, and Environmental

Public health has been redefined for the past decade, incorporating concerns that go beyond communicable diseases. Many populations suffer, for example, from endemic obesity, and it has been a major challenge for public health agencies to design strategies to fight it. Obesity is known as a multi-factorial disease caused by genetic, social and environmental factors. If data from EHRs and smart devices (e.g., Fitbit, Apple Watch) could be brought together with social and environmental data, it might be possible to study several factors relating to physical activity (or lack thereof) and obesity in a population (20). For instance, the walkability of a city and quality of the environment (e.g., air pollution, crime rates, availability of public transport, traffic, etc.) can affect the amount of exercise a person gets (20). In addition, obesity in a person's social network can be a predictor of the individual's BMI (e.g., if their friends do not go out and exercise, mostly living a sedentary lifestyle, the individual may do the same).

### Precision Public Health

As can be inferred from the previous section, many data types and use cases can be helpful both for precision medicine and public health. Further, for these data types to provide results, the datasets must include at least a large variety and volume of high-speed data, characterizing Big Data. In this section, we discuss research question (v) as we describe specific use cases that bridge these two fields, converging into precision public health with the use of Big Data. Drawing on Dolley's framework of areas in which Big Data can be applied in Precision Public Health (9), we divide our discussion into Targeted Interventions (divided into personalized risk factors and feedback loops), Risk Prediction, and Disease Surveillance. The fourth broad area that Dolley defines, Understanding Disease, is distributed throughout the first areas.

#### Targeted Interventions—Personalized Risk Factors

One of the promises of Big Data in healthcare is the discovery of new risk factors for diseases (9, 20). This is helpful in public health as the discovery of risk factors can help in the development of strategies for prevention of diseases and behavioral change in populations. Massive datasets from heterogeneous sources and including the data types described earlier in the paper allow population, subpopulation and personal-level analyses, which can lead to the discovery of personalized risk factors that take into account individual variables and traits, characterizing a precision medicine application as well.

Without Big Data, it would be extremely hard to perform stratification and detect these risk factors: for example, certain factors may be beneficial to some people but harmful to others, so the overall effect that will be measured in this population will be zero. Only with high-speed data collection and high volumes and varieties of data is there enough granularity and variety in the data to calculate such statistical interactions. In other words, only with population-level data is it possible to detect relationships that will be beneficial for a precision approach.

One interesting case of environmental data for detecting personalized risk factors is the use of Big Data to predict environmental factors, such as concentration of a certain particulate matter in the air. Identifying environmental risk factors can help determine who is more at risk of exposure (e.g., expectant mothers), allowing personalized treatment, and it can lead to targeted interventions with the goal of improving population health (e.g., implementing policies in urban environments) (78). Prediction of air pollutants is an excellent use case to illustrate how the lines between precision medicine and public health can converge. The analysis and insights gained from using Big Data to analyze environmental factors can help both individuals at a personalized level (such as detecting levels of pollution in their neighborhood) and also support targeted population-level interventions (for example, for an entire city).

#### Targeted Interventions—Feedback Loops

We have previously discussed how analytic methods applied to PGHD and social data (and possibly in conjunction with other data types, such as clinical date) are used to analyze data quickly, gaining insight into the behavior of individuals. These insights can be communicated back to the user (for example, through phone notifications) generating a feedback loop: data is collected and analyzed, and actionable insights are transmitted back to users with information about their health (and possibly even recommendations for improvement). This can greatly improve the engagement of patients with their health and, by providing suggestions for health improvements based on an individual's specific behaviors, these loops represent a case of Big Data use for precision medicine.

However, they can also be seen as a form of targeted intervention in public health by being applied to a large percentage of the population in a very specific, personalized manner. In the past, interventions to improve exposure to disease risk factors meant that doctors gave recommendations to patients (e.g., stop smoking or drinking, be physically active). With real-time feedback loops enabled by smart devices which collect and transmit data, as well as the application of advances analytics, including AI methods, these interventions can reach a much larger sample of the population in little to no time. Further, these interventions can be viewed as *precise*, as they are based on data collected at an individual level. Therefore, the same data types and uses for precision medicine can be helpful for interventions in public health, leading to precision public health. Jain et al. (47) highlights, for example, that wearable accelerometers can track functional outcomes in people with neurological disorders and provide data for designing rehabilitation programs. This can be seen as precision public health, as information contained in feedback loops can provide interventions to a subgroup of the population.

#### Predicting Risk

For medicine to be precise, it must rely on evidence-based, richly diverse data aggregated from a variety of sources. This means crossing traditional boundaries of what is thought of as health data (omics, clinical data) to aggregate and integrate social and environmental determinants of health. Integrating a multitude of data from different sources will lead to a better understanding of what makes treatment effective for a person or group of people. It will also bring precision medicine closer to public health, as social and environmental variables are usually collected, analyzed, and used at a population level. For example, Xiaonan et al. were able to estimate accurately exposure to particulate matter in the air using Google Maps data (79).

Authors such as Prosperi et al. (8) set disease prevention as one of the goals of precision medicine. One can argue that precision medicine deals with the specific traits of an individual in order to protect that person from diseases. However, once this knowledge is applied to a community of individuals with similar characteristics, precision medicine converges into precision public health. In the same vein, traditional public health analysis has typically looked at population-level data, including social, environmental, and clinical data. Including more precise data about each individual, such as omics, can improve prevention efforts, as they can be targeted to more specific subgroups of a population, and can help researchers understand the characteristics of diseases (20) better. Further, models created using this wide variety of information can be used to predict risks. As described by Dolley, early attempts to use Big Data for public health purposes with Google Flu Trends data resulted in a failure, leading researchers to integrate more data sources (9).

#### Disease Surveillance

In the previous sections, we discussed how novel sources and types of data can lead to increased disease tracking and surveillance. With the ubiquity and connectivity of new technologies, researchers can study movements, patterns, and behaviors of individuals and populations, by tracking affected individuals. The key difference between traditional approaches of surveillance and Big Data is that Big Data can lead to disease surveillance in close to real-time. As Dolley (9) suggests, “Access to huge volumes of streaming real-time data generated by humans seems at once an ideal signal repository for identifying and tracking affected individuals.”

Ginsberg et al. (80) uses clinical data (the number of physicians in a region), and social data (search queries) to estimate the probability that a visit to a physician in that region is related to influenza.

An interesting example that leverages a precision approach to public health is presented in Arora et al. (81), in which the authors divided India into several regions according to severe, moderate and mild depending on the number of COVID-19 cases and created separate models for each of the states and union territories. Unlike the COVID-19 prediction cases described above, this work has a precision approach to public health that targets detailed areas within a larger population or region.

One caveat to be made here is that disease surveillance as well as the handling of any type of patient data, need to be assessed in terms of privacy of patient data and anonymization. We will discuss these topics in the section entitled The Challenges to a Data-Driven Approach to Precision Medicine and Public Health.

## Discussion

### Convergence of Precision Medicine and Public Health into Precision Public Health

The previous section provides details on several examples of data types, sources and use cases from precision medicine and public health, as well as how these can converge into the field of precision public health. Indeed, as we add large amounts of diverse data to health studies and research, it becomes harder to differentiate between precision medicine and public health. Further, with generation and access to more data, we converge to precision public health.

For example, we cited U.S. state programs that use genomic and clinical data to screen newborns for certain conditions. These programs have been run for more than 50 years, even before the term precision medicine were used, and are part of public health tracking and interventions. However, it is possible to argue that by targeting a specific subgroup of newborns inside a population, this could be considered one of the earliest efforts in precision public health (74). In the same token, we mentioned how COVID-19 studies that leverage Big Data to study the incidence of the virus in a country could also be considered examples of precision public health. In this sense, precision public health is nothing more than the natural evolution of the fields of precision medicine and public health. With the use of Big Data, the individual and the population are complementary and health problems can be solved both at the micro and macro level.

One interesting observation in our previous definition of data types and uses is that we did not explicitly include epidemiological data, i.e., data focusing on a disease in a population, despite having extensively described studies and examples of the use of this data (82). This is owing to the fact that this data source is typically collected from other datasets and used for population-level surveillance, such as patient records and social media information.

As a further example of how Big Data can be used to gain insights into the health and behavior of individuals and applied in a population-context, we propose the following example: the Canadian Health Measures Survey (CHMS) (62) is a major survey comprised of: (i) an hour-long interview in the respondents' home; (ii) a visit to a temporary clinic to collect physical measures; and (iii) use of a fitness tracker for a week. Most of the measures in this survey, such as body composition or heart rate, can be collected using smart technologies, and the aforementioned platform can reduce social and recall biases. Incorporating smart technologies in survey design will minimize time and financial burdens of clinicians and interviewers, while data can be reported in real-time. By leveraging the data already collected from personal devices for long periods of time, studies could minimize follow-up losses by providing automated data collection while ensuring the data are more representative than those obtained from the fitness tracker.

In other words, the use of smart technologies to collect social data here would lead to more precise and accurate data on the health of individuals, which would allow public health agencies to have a more complete view of the health and behavior of these groups. Further, it would also help these agencies to create population-wide interventions in the public health sphere. This kind of bi-directional relationship between the two fields, described in detail in the previous section, is what we call the convergence of precision medicine and public health into precision public health.

Having data, however, is only one half of the equation. Research should generate insight, and the use of advanced analytics—for example through the use of AI—to allow us to make sense of and extract meaningful information from a massive amount of diverse data. We propose that, if the aggregation and integration of heterogeneous data is the door to converging precision medicine and public health into precision public health, then Big Data and its associated technologies (including AI and statistical analytic methods) is the key. It is the tool that allows us to analyze and hopefully understand what all of this data means at an individual and population level. Indeed, the studies mentioned in the Results section use a wide variety and quantity of data, combined with advanced analytic tools, to study complex interactions on the health and behavior of individuals. However, achieving precision in the context of public health is not a trivial task.

Researchers and policy makers must be very careful, as improving an individual's health does not always translate to an improvement in the health of an entire population. For example, a prediction model that can improve the health of a large sample of the population can still neglect minorities (83). Here, it is imperative that evidence-based research is used by public health agencies and policy makers to design effective treatments and interventions for communities and populations. While this may seem like an obvious statement, in practice it is difficult to integrate knowledge translation strategies with public health. Fafard and Hoffman (84) state that while public health policies must be based on the best available evidence, “all too often efforts to do so rely on mechanistic and unrealistic views of the process by which public policy is made.” Indeed, while knowledge translation was successful in health care areas such as clinical practices, the same success was not achieved for public health. A systematic review of knowledge translation strategies (85) found, for example, that multifaceted knowledge translations strategies may lead to a change in knowledge but not practice, and no singular knowledge translation strategy was effective in all situations. Rather, strategies should be applied depending on the stakeholders involved and the kind of knowledge being used.

Further, while comprehensive data that allows for precision healthcare across populations is ideal, it is also important to note also that data monitoring, collection, analysis and dissemination is expensive and time-consuming. Therefore, while aggregation and integration of heterogeneous data types and sources are necessary, researchers should try to balance that with real-world demands to obtain the best results (8).

In addition to concerns regarding knowledge translation and research limitations, we must also consider challenges to a data-driven approach to precision public health in the context of data generation. These challenges are described in the next section.

### The Challenges to a Data-Driven Approach to Precision Medicine and Public Health

In this section, we briefly state some of the current challenges in healthcare that prevent the generation of data needed at the scale, volume, and velocity necessary for Big Data.

The first and most important challenge is data fragmentation. Currently, data is fragmented throughout current EHR systems. In addition, providers have systems that are often not interoperable. Coupled with a lack of comprehensive formats and standards for data storage, these factors lead to great difficulty in data sharing. In practice, this means that patients interact with different providers throughout their lives while they access care services and end up losing access to past data (8, 41, 86).

Ultimately, patients' health data end up in silos and cannot be integrated with data from other providers or sources, such as connected devices (e.g., smartphones, fitness trackers). This means that there is no easy way to obtain a holistic view of a patient's health, leading to errors, delays, and poorer health outcomes (87). This also limits the availability of massive datasets needed for precision public health.

There are also several barriers to the availability of massive genomics datasets, including security and privacy concerns, prohibitive individual costs, rights to ownership of genomic data, and data sharing. The latter is related to a lack of interoperability among systems that store genomics data. Considering that a human genome generates over 200 gigabytes of data and that estimates predict that more than 100 million genomes will be sequenced by 2025, storage and network transfer speeds also limit data sharing (17, 67, 88).

The question of data accuracy must also be addressed when dealing with large datasets from heterogeneous sources. As mentioned, many authors extend the 3 Vs of Big Data to include additional properties, one of which is Veracity. For example, social media data may be biased regarding age, and also concerning minority groups or those who live in remote/rural locations may be underrepresented owing to limited Internet access (67). Conclusions drawn from social media datasets should also be assessed very carefully as many of the results reported in the literature have not been repeatable.

Johnson et al. defines three major problems in data collection and cleaning in healthcare and clinical settings: compartmentalization, corruption, and complexity (40). While not in the scope of this paper, it is interesting to note that Big Data and Artificial Intelligence techniques may be able to solve some problems related to corruption (e.g., estimating values of missing data based on time-series analysis) and complexity (e.g., through prediction and state estimation). Johnson et al. provides a good overview of techniques in this context (40).

Currently, Big Data and AI are often mentioned together and seen as complementary. In short, Big Data techniques and associated technologies (including storage and analytic engines, data warehouses, pre-processing and visualization tools, among others) can enable Artificial Intelligence algorithms to access, process and analyze the data (67) efficiently. O'Leary described how AI contributes to at least the basic 3 Vs of Big Data, as it can aid in the processing of large volumes of data, increase the speed in which data is analyzed and shared, and process a variety of data (both structured and unstructured) (89). Precision public health, owing to its dependency on insights generated by the analyses of Big Data, depend on the quality of the data and in the models developed to interpret this data. Therefore, it is interesting to explore potential limitations of these models.

One of the issues is the quality of data used to create and train (or “teach”) an AI model. For example, if the data is biased, the program may make skewed decisions when outputting information (90). As an example, consider a security system that uses AI to analyze the face of a person and check whether that person is authorized. If the data set used to train this system does not have high quality, varied data (e.g., if it is comprised mostly of men's faces), the learning process could potentially be affected. In the example of a men's face data set, for instance, the software could produce unfortunate results when trying to analyze the faces of women (67, 91). Extending this problem to precision medicine and public health, as stated in the previous section, researchers need to be very careful—especially in dealing with precision public health—that the models are not biased and do not exclude any segment of the population that is relevant or provide misleading insights.

In addition, security of these models is also a concern. Adversarial attacks, for example, happen when an input to a model is specifically designed by an attacker to cause perturbations to the model and cause them to make a mistake (92). This is dangerous in several situations, and in healthcare it can lead to grave consequences. In theory, it would be possible for an attacker to falsify a certain output, such as a diagnosis or a prescription. Current research also extends to prevent adversarial attacks, for example by training the model on possible examples of adversarial inputs (92).

Another challenge is the problem of explainability in AI algorithms, or in other words, how the AI algorithm arrived at the conclusion it did. In other words, how can we identify how an AI algorithm arrived at the conclusion it did? And if we cannot identify this, how can we trust the predictions of an AI model? The issue of explainability can be exemplified when we consider neural networks (e.g., Deep Learning). In short, neural networks are AI algorithms that try to achieve a prediction by creating models that simulate the operation of the human brain. Neural networks work as black-boxes, in the sense that it is not possible to describe how the algorithm reached specific conclusions. In this technique, small computational units (the “neurons”) are arranged in layers that are connected (digital “synapses”) (91). Each unit in the layer processes data and each layer integrates input from previous layers. At the end of the process, the topmost layer provides an output. These algorithms learn through an iterative method; in neural networks, the learning process simulates the human brain by conditioning the “neurons.”

A Machine Learning researcher gives the following example (91): “Where is the first digit of your phone number stored in your brain? Probably in a bunch of synapses, probably not too far from the other digits. But there is no well-defined sequence of bits that encodes the number.” Therefore, understanding the explainability of a neural network algorithm is significant, and this lack of transparency is what gives the impression of a “black-box.”

If researchers are not able to understand how an algorithm arrived precisely at its prediction, it becomes difficult to place trust in AI systems. In simple use cases, it is easy to verify the accuracy of AI (e.g., by looking at a picture, we can tell if the animal in the picture is a dog, even if the algorithm classified it as a cat). However, the situation becomes more complicated as we increase the complexity of the decision. If a neural network analyses clinical data and concludes that a patient has cancer, is it possible to trust this diagnosis if we do not understand how the machine arrived at it? On the one hand, the neural network may have recognized patterns and features that humans do not have the capacity to analyze; on the other hand, the algorithm could simply be wrong. Should we trust machines in the same way that we apparently trust our doctors even though they also may be wrong in making a diagnosis? Further, if we do not understand yet how our own brains work, should we be concerned about understanding how an AI system's reasoning takes place?

To solve this issue, the field of explainable AI has experienced rapid growth in the last years, as it seeks to improve transparency and justify decision making based on the results of AI models. Arrieta et al. provides the following definition of explainable AI: “Given an audience, an explainable Artificial Intelligence is one that produces details or reasons to make its functioning clear or easy to understand” (93), and provides an overview of current terminology and techniques for explainable AI. Advances in this field can greatly improve the trust and human factor challenges and contribute to an increasing adoption of a data-driven approach to precision public health.

In addition, it is also important to note that the problems mentioned here are not specifically unique to AI but can be present in statistical techniques and modeling in general, even if they are not using Big Data or AI algorithms (90). As previously discussed, the quality of an algorithm's results, regardless of whether it uses AI techniques or not, will depend on the quality of the input data and the biases of the model.

Nevertheless, since AI can provide powerful prediction tools and is becoming increasingly popular in the field of healthcare, these considerations of data quality and trust can hamper the acceptance of such techniques, particularly in the context of predicting health outcomes and diagnosis. Research in areas such as explainable AI can help to minimize these issues and increase the interpretability of AI algorithms (67). Finally, a data-driven approach to precision public health has to overcome societal challenges, such as religious beliefs, political views, science denial, and racial disparity (8).

### Security and Privacy of Personally Identifiable Information (PII)

Two major challenges that must be addressed when dealing with large datasets comprised of sensitive information are security and privacy. Generally, different jurisdictions contain acts that protect PII. In Canada, for example, The Protection and Electronic Documents Act (PIPEDA) regulates the collection, use and disclosure of PII for private sector organizations involved in a commercial activity. This federal act applies to all types of PII (94, 95). Several provinces have adopted health sector laws dealing with personal *health* information (PHI), some of which are deemed substantially similar to PIPEDA and taking precedence in these provinces (94–96). PIPEDA's principle of Safeguards mandate that PII “be protected by security safeguards appropriate to the sensitivity of the information,” including encryption, authentication, and access control (95). It is important to note that organizations must obtain informed consent for the collection, use and disclosure of PII and state their purposes of data collection (95). The Health Insurance Portability and Accountability Act (HIPAA), which applies to subsets of health custodians in the U.S., offers a similar but more comprehensive list of technical, physical and administrative safeguards (97). The General Data Protection Regulation in Europe provides a more comprehensive framework than the previously cited regulations for the protection of personal information, including the right of access by the data subject and the right to be forgotten (98).

Ultimately, this means that the collection of data from individuals which is accounted for in regulatory acts such as the ones just described must protect this information using different kinds of safeguards, considering cybersecurity attacks and taking the necessary measures to prevent it. This would include, for example, mobile apps collecting social data (99).

The collection, use and disclosure of PII for research are typically not subject to regulatory acts but must get approval from review ethics boards, which also require safeguards according to the sensitivity of data (94, 100).

One way to deal with PII is to anonymize it, meaning that the datasets will be de-identified so that data will not be considered identifiable; as such, it can be freely shared and stored (101).

To anonymize datasets, direct identifiers (DIs) in the dataset are removed. In addition, quasi-identifiers can be masked to decrease the risk of re-identification. For example, the last 3 digits of a postal code can be removed, as well as the day and month in the date of birth in a database (101).

In the context of precision public health, we are dealing with massive datasets with information collected from different sources. Therefore, after a large dataset with diverse data from heterogeneous sources is anonymized, a challenge becomes how to link records from different sources. Two possible methods include Direct Linkage and Probabilistic Record Linkage.

- Direct Linkage: this involves methods that include the use of a unique identifier for record linkage followed by the removal of DIs to anonymize data (101–104). For example, the Institute for Clinical Evaluative Sciences (ICES) repository contains several types of health administrative data. New data typically contains Ontario health card numbers, which are used to generate an ICES-specific key number (IKN) that uniquely identifies individuals and links records from different datasets (102).

The BORN registry, which includes all births in Ontario, follows a somewhat similar approach in which DIs are directly used for record linkage. These identifiers are not removed but converted to a pseudonym. While this can simplify linkage, pseudonyms are still considered PII and subject to regulations; in addition, their use increases the risk of re-identification (101, 105).

In these examples, DIs are used to link records belonging to the same individual directly. This is called deterministic record linkage (102).

- Probablistic Record Linkage (PRL): In several situations, incoming data may not contain DIs. In this case, PRL is used for record linkage, which considers similarities and frequencies of quasi-identifiers in a dataset to predict the probability of a record belonging to one person (e.g., a rare name in the population of study in two records increases the probability that the records belong to the same individual) (101).

In the case of ICES, incoming data may not contain health card numbers. The Ministry of Health's Registered Persons Database (RPDB), containing demographic information on Ontario residents, is used for PRL. Once a new record is identified, the health card number of the individual in the RPDB is used for IKN generation and to link this record deterministically to others in the ICES repository, followed by the removal of DIs (101).

The Centre for Health Services and Policy Research at the University of British Columbia also applied PRL for linking records. Researchers created a master file of all provincial health service recipients, and different combinations of direct and quasi-identifiers were used for PRL depending on which variable was available in a given record (e.g., sex, birth year, Personal Health Number) (101, 104).

One challenge with this method is that links between records can be uncertain. In the previous example, researchers filtered possible links according to similar patterns of agreement and manually reviewed them for inclusion. The reviewers took a conservative approach to identify matching records, as minimizing the number of false positives was a priority for subsequent researches involving the databases (104).

The reason why privacy of PII is so important is that, as we explained throughout this paper, for precision public health to improve the health of individuals and populations, generation of Big Data is essential. Large volumes of linked, accurate and representative data collected at an efficient and fast pace must be present to generate actionable insights. However, privacy concerns may lead to hesitancy on the part of individuals to share their data with researchers and public health agencies. This is particularly true of social data and PGHD. In this way, privacy regulations must be respected and, in case the data is anonymized, appropriate precautions must be taken to diminish the risk of re-identification while appropriate techniques for data linkage must be used to ensure that information can be linked across a wide variety of data types and sources. This, in turn, will facilitate state-of-the-art research into the field of precision public health.

As previously mentioned, knowledge translation is an essential part of translating actionable insights into practice, specially in public health interventions. The dimension of privacy must also be considered when creating and deploying knowledge translation strategies. A report from the Canadian Marketing Association reports that, while consumers in Canada, the U.K. and the United States are becoming more comfortable sharing data with companies, more control over what data are currently being collected by organizations is desirable, and trust is essential in this matter (106). Another study shows that patients wish to be consulted before de-identified medical records are used for research (107). In this manner, researchers must ensure that individuals that generate data are aware that their data are being used for research and that all appropriate safeguards and security measures are being taken to handle these data. In addition to collecting data in an ethical, secure and private manner, this will also garner public support for precision public health initiatives.

### Critiques to the Field of Precision Public Health

In addition to the challenges mentioned above, there has been recent critiques of the field of precision public health. Chowkwanyun et al. (108) points out that the term precision public health seems to have two different definitions within the science community. The first, more restrictive view, overwhelmingly focuses on the use of genomic data. This data type is used to create subgroups in the population that can be targeted for specific interventions according to their unique genetic traits. The second, broader definition encompasses the use of vast amounts of novel data, such as the data types discussed throughout this paper (including, but not limited to, genomics) in order to gain actionable insights into the health of populations with a much more detailed and precise focus for interventions. Given these views, Chokwanyun et al. (108) have two main critiques: (i) the first definition, focusing solely on genomic data, risks ignoring a plethora of data types and socio-economic factors that can contribute to our understanding of population health. In addition, despite advances in its generation and analyses, genomics currently does not fulfill its promises of revolutionary insights into our health. The focus on genomics, according to the authors, seems to be the more prevalent definition when discussing precision public health; (ii) while Chokwanyun et al. (108) recognize that recent innovations (e.g., in sensing technologies, computational power and data storage) can improve our understanding of individual and population health, the authors believe that adding the word precision to public health is merely a “rebranding” exercise. Rather, traditional public health always made use of tools and technologies, and the novel data types described in this paper and associated technologies are simply additional tools to help the field achieve its goal of improving population health.

On the other hand, authors such as Horton (109) support the field of precision public health, stating that researchers in this emerging field do not seek to ignore determinants of health, but rather to gain a new understanding of them through the use of new data types and technologies. The goal of the field is not to focus on genomics to the exclusion of everything else, but to make use of all available data in order to improve population health—which is the ultimate goal of traditional public health efforts. However, Horton (109) does not address the previous concerns of many researchers having an over-emphasis on genomic data (110).

Khoury, the founding director of the Office of Genomics and Precision Public Health of the CDC (74), argues that while many critics of precise approaches to medicine and health equate this term with genomics, the goal of precision public health is much more in line with the second definition mentioned by Chokwanyun et al.: using all available data, coupled with advanced analytics and technologies, to improve population health (108). Traditional medicine and public health have always been complementary—medicine focuses on the individuals through traditional health care, while public health focuses on addressing socio-environmental causes of declining health in populations. By improving the individual, the quality of life of populations is also improved; conversely, by addressing the community, the quality of life of the individual increases. In this context, precision publichealth is about making interventions in the community more precise—for example, by understanding what groups in a population might be more affected by a certain cause and providing a targeted approach to solve the issue. While the idea of targeted interventions in the population is not new, new technologies and data (as seen throughout this paper) allows public health practitioners, for the first time, to understand and improve population health with much more detail and precision than ever before—for instance using genomic data to identify anomalies in a population; social data to study subgroups of Facebook or Twitter users; or environmental data to look at the prevalence of a condition in a neighborhood. All these examples have in common the idea that novel data types and technologies allow us to target a much narrower, precise segment of a community to better understand its health.

Throughout this paper, we explored potential data types, their uses and sources, and how they can contribute to a data-driven approach to precision public health. While the risk of relying too much on genomic data is certainly worrisome, and researchers and agencies need to be cautious of not focusing too much on one area that they “miss the forest for the trees,” we believe that precision public health—meaning the collection and analyses of all available data, enabled by advances in sensing, analysis and storage technologies, to understand and improve the health of individuals and populations better through targeted interventions—represent a new era in public health efforts. We discussed potential use cases in precision public health, from targeted interventions to risk prediction and surveillance. While these have always been the priority of public health, the use of precise methods that “use the best available data to target more effectively and efficiently interventions of all kinds” (109) can greatly revolutionize our understanding on the health of individuals, populations, and the relationship between micro and macro levels of health. In this sense, precision public health can be seen as a new era in the practice of public health and precision medicine; it is the natural combination of these two fields to improve how we understand our health and the health of the community around us, made possible by the generation and use of Big Data.

## Conclusion

Recent advances and emerging technologies are resulting in an explosion of rich and diverse health data sets. This, in turn, is causing a paradigm shift in healthcare, bringing together the fields of precision medicine and public health and converging them into precision public health. Aggregation, integration, and analysis of Big Data are key, bolstered by advanced analytical methods including Artificial Intelligence algorithms, as it can help researchers make sense of available data and their interactions.

In this paper, we have conducted a survey and highlighted some of the key data types and uses of these data currently being studied for precision medicine and public health through a review. These data types include -omics, clinical, social, patient-generated, environmental, and demographic data. We also included examples of precision medicine and public health that use these data types, alone or in combination. Using the taxonomy of data types in this paper, and classification of various studies, we intend to show that with the aggregation and integration of diverse data types from several sources, the lines between what constitutes precision medicine and public health become blurred. Indeed, several examples of precision medicine and public health can be classified into precision public health with the use of novel technologies and more data. Furthermore, these fields can complement each other and provide precision medicine doctors and public health practitioners with new insights into individual and population-level data, leading to the field of precision public health—with the potential of revolutionizing traditional public health by allowing a more precise and targeted approach to improving the health of populations, through the use of new technologies.

Further, we discussed several challenges that exist in healthcare and computer science that currently prevent a complete data-driven approach to healthcare. Among them, we can cite complexity of data, interoperability issues among healthcare providers, lack of provenance (which, in turn, results in a lack of trust) in AI systems and difficulty in knowledge translation approaches. The major challenges of security and privacy of PII were also discussed, and how they contribute or hinder precision public health. We also discussed novel research that can solve some of these challenges, including explainable AI, record linkage in large datasets and anonymization. We finish with an overview of current critiques to the field of precision public health, including the fact that the “precision” label is nothing more than a rebranding of traditional public health efforts, now bolstered by novel technologies. It is our view that the alignment of precision medicine with its focus on individuals, and of public health with the goal of improving the health of populations, enabled by the sudden explosion of Big Data and associated technologies for processing, storaging, and analyzing of data, enable a revolution in the way that researchers and agencies understand and improve health, both at the individual and the population-level.

Limitations in our review include the fact that our initial goal was to study analysis techniques rather than focusing on uses of data. Most papers we found were related to health care rather than computer science, and were more qualitative than quantitative (e.g., discussing uses of data rather than techniques or technical details). In addition, since so many of the terms used here do not have a concise definition across research (e.g., personalized medicine is sometimes used interchangeably with precision medicine), it is possible that, despite our methodology, important papers related to data types, analysis, and use were missed.

Future work should focus on exploring each data type separately to identify more use cases and opportunities for bridging the gap between precision medicine and public health. In addition, reviews focused solely on computer science databases could yield more information on aggregation, integration, and analysis techniques for Big Data health analytics.

## Author Contributions

PV and TB were responsible for conducting the literature review, summarizing the results, and discussing the findings. A preliminary version of this manuscript was produced as a final report on a graduate course on software engineering for big data directed by PA. PM, PA, and DC provided direction to the manuscript's writing and preparation. All authors contributed to the article and approved the submitted version.

## Conflict of Interest

The authors declare that the research was conducted in the absence of any commercial or financial relationships that could be construed as a potential conflict of interest.

## References

[1] Adams SA Petersen C. Precision medicine: opportunities, possibilities, and challenges for patients and providers. J Am Med Inform Assoc. (2016) 23:787–90. 10.1093/jamia/ocv215 26977101 PMC9396673

[2] MedlinePlus. What Is Precision Medicine? Available online at: https://medlineplus.gov/genetics/understanding/precisionmedicine/definition/ (accessed November 30, 2020).

[3] Khoury MJ. The Shift From Personalized Medicine to Precision Medicine and Precision Public Health: Words Matter!. Centers for Disease Control and Prevention. (2016). Available online at: https://blogs.cdc.gov/genomics/2016/04/21/shift/ (accessed August 8, 2019).

[4] Juengst ET McGowan ML. Why does the shift from “personalized medicine” to “precision health” and “wellness genomics” matter? AMA J Ethics. (2018) 20:E881–90. 10.1001/amajethics.2018.881 30242820

[5] What is Public Health? CDC Foundation. Available online at: https://www.cdcfoundation.org/what-public-health (accessed August 8, 2019).

[6] Weeramanthri TS Dawkins HJS Baynam G Bellgard M Gudes O Semmens JB. Editorial: precision public health. Front Public Heal. (2018) 6:3–5. 10.3389/fpubh.2018.00121 PMC5937027 29761096

[7] Khoury MJ. Precision Public Health and Precision Medicine: Two Peas in a Pod. Blogs. CDC. Available online at: https://blogs.cdc.gov/genomics/2015/03/02/precision-public/ (accessed August 29, 2019).

[8] Prosperi M Min JS Bian J Modave F. Big data hurdles in precision medicine and precision public health. BMC Med Inform Decis Mak. (2018) 18:1–15. 10.1186/s12911-018-0719-2 30594159 PMC6311005

[9] Dolley S. Big data's role in precision public health. Front Public Heal. (2018) 6:68. 10.3389/fpubh.2018.00068 PMC5859342 29594091

[10] Leff DR Yang GZ. Big data for precision medicine. Engineering. (2015) 1:277–9. 10.15302/J-ENG-2015075

[11] Catalyst. Smartphone Behaviour in Canada and the Implications for Marketers in 2016 - Catalyst. Catalyst (2016). Available online at: http://catalyst.ca/2016-canadian-smartphone-behaviour/ (accessed October 12, 2018).

[12] Medeiros H. 57% da População Brasileira usa Smartphone, diz Estudo. EXAME. EXAME. (2016). Available online at: https://exame.abril.com.br/tecnologia/57-da-populacao-brasileira-usa-smartphone-diz-estudo/ (accessed October 12, 2018).

[13] Global Smartwatch Unit Sales 2014-2018. Statistic. Statista (2018). Available online at: https://www.statista.com/statistics/538237/global-smartwatch-unit-sales (accessed October 12, 2018).

[14] Nielsen. Connected Life: Canadian Trends (2015).

[15] Electronic Health Records. Canada Health Infoway. Available online at: https://www.infoway-inforoute.ca/en/solutions/digital-health-foundation/electronic-health-records (accessed September 27, 2018).

[16] Embleema. Embleema WhitePaper. (2018). Available online at: https://icocube.io/uploads/Embleema.pdf

[17] Grishin D Obbad K Estep P Cifric M Zhao Y Church G. Blockchain-Enabled Genomic Data Sharing and Analysis Platform. (2018). Available online at: https://www.nebulagenomics.io/assets/documents/NEBULA_whitepaper_v4.52.pdf

[18] Berry D. There is Nothing More Personal Than Your Genome. Tedx Talks. Available online at: https://www.youtube.com/watch?v=M3SLHhWYxiY (accessed November 12, 2018).

[19] Kulemin N Popov S Gorbachev A. The Zenome Project: Whitepaper Blockchain-Based Genomic Ecosystem. Zenome (2017). Available online at: https://neironix.io/documents/whitepaper/657/whitepaper.pdf

[20] Barrett MA Humblet O Hiatt RA Adler NE. Big data and disease prevention: from quantified self to quantified communities. Big Data. (2013) 1:168–75. 10.1089/big.2013.0027 27442198

[21] Sakr S Elgammal A. Towards a comprehensive data analytics framework for smart healthcare services. Big Data Res. (2016) 4:44–58. 10.1016/j.bdr.2016.05.002

[22] Sivarajah U Kamal MM Irani Z Weerakkody V. Critical analysis of Big Data challenges and analytical methods. J Bus Res. (2017) 70:263–86. 10.1016/j.jbusres.2016.08.001

[23] Ramaswami R Bayer R Galea S. Precision medicine from a public health perspective. Annu Rev Public Health. (2018) 39:153–68. 10.1146/annurev-publhealth-040617-014158 29166244

[24] Levac D Colquhoun H O'Brien KK. Scoping studies: advancing the methodology. Implement Sci. (2010) 5:69. 10.1186/1748-5908-5-69 20854677 PMC2954944

[25] PubVenn. PubVenn Search - Personalized Medicine and Precision Medicine (2021).

[26] MedlinePlus. What Is the Difference Between Precision Medicine and Personalized Medicine? What About Pharmacogenomics? (2020).

[27] Ward SL. ‘Omics, Bioinformatics, Computational Biology. AltTox (2014). Available online at: http://alttox.org/mapp/emerging-technologies/omics-bioinformatics-computational-biology/#:~:text=Thissection describes emerging technologies,make sense of the data

[28] Mougin F Auber D Bourqui R Diallo G Dutour I Jouhet V. Visualizing omics and clinical data: which challenges for dealing with their variety? Methods. (2018) 132:3–18. 10.1016/j.ymeth.2017.08.012 28887085

[29] Wu PY Cheng CW Kaddi CD Venugopalan J Hoffman R Wang MD. Omic and electronic health record big data analytics for precision medicine. IEEE Trans Biomed Eng. (2017) 64:263–73. 10.1109/TBME.2016.2573285 27740470 PMC5859562

[30] Institute NHGR. Fact Sheets About Genomics. (2020). Available online at: https://www.genome.gov/about-genomics/fact-sheets

[31] Savage N. Calculating disease. Nature. (2017) 550:S115–7. 10.1038/550S115a 29045372

[32] Andreu-Perez J Poon CCY Merrifield RD Wong STC Yang G. Big data for health. IEEE J Biomed Heal Informatics. (2015) 19:1193–208. 10.1109/JBHI.2015.2450362 26173222

[33] Behjati S Tarpey PS. What is next generation sequencing? Arch Dis Child Educ Pract Ed. (2013) 98:236–8. 10.1136/archdischild-2013-304340 23986538 PMC3841808

[34] Illumina. Deep Sequencing. Available online at: https://www.illumina.com/science/technology/next-generation-sequencing/plan-experiments/deep-sequencing.html#:~:text=Deepsequencing refers to sequencing,1%25 of the original sample

[35] Thaker VV. Genetic and epigenetic causes of obesity. Adolesc Med State Art Rev. (2017) 28:379–405. 30416642 PMC6226269

[36] Bardakjian T Gonzalez-Alegre P. Towards precision medicine. Handb Clin Neurol. (2018) 147:93–102. 10.1016/B978-0-444-63233-3.00008-7 29325630

[37] Azuaje F. Artificial intelligence for precision oncology: beyond patient stratification. NPJ Precis Oncol. (2019) 3:1–5. 10.1038/s41698-019-0078-1 30820462 PMC6389974

[38] Tsimberidou AM Eggermont AMM Schilsky RL. Precision cancer medicine: the future is now, only better. Am Soc Clin Oncol Educ B. (2014) 34:61–9. 10.14694/EdBook_AM.2014.34.61 24857061

[39] Rich J. Library Guides: Data Resources in the Health Sciences: Clinical Data. Available online at: http://guides.lib.uw.edu/hsl/data/findclin (accessed August 9, 2019).

[40] Johnson AEW Ghassemi MM Nemati S Niehaus KE Clifton D Clifford GD. Machine learning and decision support in critical care. Proc IEEE. (2016) 104:444–66. 10.1109/JPROC.2015.2501978 27765959 PMC5066876

[41] Agarwala V Khozin S Singal G O'Connell C Kuk D Li G. Real-world evidence in support of precision medicine: clinico-genomic cancer data as a case study. Health Aff. (2018) 37:765–72. 10.1377/hlthaff.2017.1579 29733723

[42] Gulshan V Peng L Coram M Stumpe MC Wu D Narayanaswamy A. Development and validation of a deep learning algorithm for detection of diabetic retinopathy in retinal fundus photographs. JAMA. (2016) 316:2402–10. 10.1001/jama.2016.17216 27898976

[43] Dhillon A Majumdar S St Hilaire M El-Haraki A. A mobile complex event processing system for remote patient monitoring. Proc - 2018 IEEE Int Congr Internet Things, ICIOT 2018 - Part 2018. IEEE World Congr Serv. (2018). 180–3. 10.1109/ICIOT.2018.00034

[44] What Is Social Data? Available online at: https://www.investopedia.com/terms/s/social-data.asp (accessed August 9, 2019).

[45] Deiner MS Lietman TM McLeod SD Chodosh J Porco T. Surveillance tools emerging from search engines and social media data for determining eye disease patterns. JAMA Ophtalmol. (2016) 134:1024–30. 10.1001/jamaophthalmol.2016.2267 27416554 PMC5227006

[46] Reece AG Danforth CM. Instagram photos reveal predictive markers of depression. EPJ Data Sci. (2017) 6:15. 10.1140/epjds/s13688-017-0118-4

[47] Jain SH Powers BW Hawkins JB Brownstein JS. The digital phenotype. Nat Biotechnol. (2015) 33:462–3. 10.1038/nbt.3223 25965751

[48] Odlum M Yoon S. What can we learn about the ebola outbreak from tweets? Am J Infect Control. (2015) 43:5630571. 10.1016/j.ajic.2015.02.023 26042846 PMC4591071

[49] HealthKit- Apple Developer. Available online at: https://developer.apple.com/healthkit/ (accessed October 12, 2018).

[50] Apple. ResearchKit and CareKit - Apple. Apple (2018). Available online at: https://www.apple.com/researchkit/ (accessed October 12, 2018)

[51] Bot BM Suver C Chaibub Neto E Kellen M Klein A Bare C. The mPower study, Parkinson Disease Mobile Data Collected Using ResearchKit Open Subject Categories Background & Summary. (2016). p. 1–9. Available online at: https://github.com/Sage-Bionetworks/mPower 10.1038/sdata.2016.11 PMC4776701 26938265

[52] Google Fit. Google Developers. (2018). Available online at: https://developers.google.com/fit/ (accessed October 12, 2018).

[53] Google Developers Blog: What's New With Google Fit: Distance Calories Meal Data and New Apps and Wearables. Google Developers (2015). Available online at: https://developers.googleblog.com/2015/06/whats-new-with-google-fit-distance.html?utm_source=ausdroid.net (accessed October 12, 2018).

[54] Shephard RJ. The Objective Monitoring of Physical Activity: Contributions of Accelerometry to Epidemiology, Exercise Science and Rehabilitation. Cham: Springer (2016). p. 383.

[55] Bunn JA Navalta JW Fountaine CJ Reece JD. Current state of commercial wearable technology in physical activity monitoring 2015-2017. Int J Exerc Sci. (2017) 11:503–15. 10.70252/NJQX2719 PMC5841672 29541338

[56] Sushames A Edwards A Thompson F McDermott R Gebel K. Validity and reliability of fitbit flex for step count, moderate to vigorous physical activity and activity energy expenditure. PLoS ONE. (2016) 11:e0161224. 10.1371/journal.pone.0161224 27589592 PMC5010194

[57] Ecobee. Smart Home Technology. Available online at: https://www.ecobee.com/ (accessed April 29, 2019).

[58] Donate Your Data. Ecobee. Smart Home Technology. Available online at: https://www.ecobee.com/donateyourdata/ (accessed April 29, 2019).

[59] Bublitz FM Oetomo A Sahu KS Kuang A Fadrique LX Velmovitsky PE. Disruptive technologies for environment and health research: an overview of artificial intelligence, blockchain, and internet of things. Int J Environ Res Public Health. (2019) 16:1–24. 10.3390/ijerph16203847 31614632 PMC6843531

[60] Time. Commuting Is Bad for Your Body and Health. Time. Available online at: https://time.com/9912/10-things-your-commute-does-to-your-body/

[61] Connelly LM. Demographic data in research studies. MedSurg Nurs. (2013) 22:269–70. 24147328

[62] Canadian Health Measures Survey (CHMS). Available online at: https://www.statcan.gc.ca/eng/survey/household/5071 (accessed July 26, 2019).

[63] Hicks JL Althoff T Sosic R Kuhar P Bostjancic B King AC. Best practices for analyzing large-scale health data from wearables and smartphone apps. NPJ Digit Med. (2019) 2:45. 10.1038/s41746-019-0121-1 31304391 PMC6550237

[64] Gordis L. Epidemiology. 5th ed. New York, NY: Elsevier Ltd (2014).

[65] Tumor DNA Sequencing in Cancer Treatment. New York, NY: National Cancer Institute. Available online at: https://www.cancer.gov/about-cancer/treatment/types/precision-medicine/tumor-dna-sequencing

[66] Chen J Liu Q Fan R Han H Yang Z Cui W. Demonstration of critical role of GRIN3A in nicotine dependence through both genetic association and molecular functional studies. Addict Biol. (2019) 25:e12718. 10.1111/adb.12718 30741440

[67] Benke K Benke G. Artificial intelligence and big data in public health. Int J Environ Res Public Health. (2018) 15:2796. 10.3390/ijerph15122796 30544648 PMC6313588

[68] Ghassemi MM Richter SE Eche IM Chen TW Danziger J Celi LA. A data-driven approach to optimized medication dosing: a focus on heparin. Intensive Care Med. (2014) 40:1332–9. 10.1007/s00134-014-3406-5 25091788 PMC4157935

[69] Nemati S Lehman LWH Adams RP. Learning outcome-discriminative dynamics in multivariate physiological cohort time series. Proc Annu Int Conf IEEE Eng Med Biol Soc EMBS. (2013) 2013:7104–7. 10.1109/EMBC.2013.6611195 24111382

[70] Looking for a Running Buddy? 6 Awesome Running Apps to Find Support. Polar Blog. Available online at: https://www.polar.com/blog/running-buddy-awesome-running-apps/ (accessed August 15, 2019).

[71] iPrescribe Exercise - Exercise Health Chronic Disease. Available online at: https://iprescribeexercise.com/ (accessed August 15, 2019).

[72] Yog:, Run With Friends Wherever They Live,. Available online at: https://thenextweb.com/apps/2012/11/14/tnw-pick-of-the-day-yog-lets-runners/ (accessed August 15, 2019).

[73] Chimmula VKR Zhang L. Time series forecasting of COVID-19 transmission in Canada using LSTM networks. Chaos Solitons Fractals. (2020) 135:109864. 10.1016/j.chaos.2020.109864 32390691 PMC7205623

[74] Khoury MJ. Reflections on Precision Public Health. Division of Public Health Information Dissemination Center for Surveillance, Epidemiology, and Laboratory Services. CDC (2018). Available online at: https://www.cdc.gov/genomics/about/file/print/Khoury-reflection_2018_508.pdf

[75] Ayyoubzadeh SM Ayyoubzadeh SM Zahedi H Ahmadi MR Niakan Kalhori S. Predicting COVID-19 incidence through analysis of google trends data in iran: data mining and deep learning pilot study. JMIR Public Heal Surveill. (2020) 6:e18828. 10.2196/18828 32234709 PMC7159058

[76] Qin L Sun Q Wang Y Wu KF Chen M Shia BC. Prediction of number of cases of 2019 novel coronavirus (COVID-19) using social media search index. Int J Environ Res Public Health. (2020) 17:1–14. 10.3390/ijerph17072365 32244425 PMC7177617

[77] Ram S Zhang W Williams M Pengetnze Y. Predicting asthma-related emergency department visits using big data. IEEE J Biomed Heal Informatics. (2015) 19:1216–23. 10.1109/JBHI.2015.2404829 25706935

[78] Zhu W Wang J Zhang W Sun D. Short-term effects of air pollution on lower respiratory diseases and forecasting by the group method of data handling. Atmos Environ. (2012) 51:29–38. 10.1016/j.atmosenv.2012.01.051

[79] Yu X Stuart AL Liu Y Ivey CE Russell AG Kan H. On the accuracy and potential of Google Maps location history data to characterize individual mobility for air pollution health studies. Environ Pollut. (2019) 252:924–30. 10.1016/j.envpol.2019.05.081 31226517

[80] Ginsberg J Mohebbi MH Patel RS Brammer L Smolinski MS Brilliant L. Detecting influenza epidemics using search engine query data. Nature. (2009) 457:1012–4. 10.1038/nature07634 19020500

[81] Arora P Kumar H Panigrahi BK. Prediction and analysis of COVID-19 positive cases using deep learning models: a descriptive case study of India. Chaos Solitons Fractals. (2020) 139:110017. 10.1016/j.chaos.2020.110017 32572310 PMC7298499

[82] Mullner RM. Epidemiology - Sources of Epidemiological Data | Britannica. Britannica. Available online at: https://www.britannica.com/science/epidemiology/Sources-of-epidemiological-data (accessed December 27, 2020).

[83] Khoury MJ Galea S. Will precision medicine improve population health? JAMA. (2016) 316:1357. 10.1001/jama.2016.12260 27541310 PMC6359904

[84] Fafard P Hoffman SJ. Rethinking knowledge translation for public health policy. Evid Policy A J Res Debate Pract. (2020) 16:165–75. 10.1332/174426418X15212871808802

[85] Larocca R Yost J Dobbins M Ciliska D Butt M. The effectiveness of knowledge translation strategies used in public health: a systematic review. BMC Public Health. (2012) 12:1. 10.1186/1471-2458-12-751 22958371 PMC3532315

[86] McGhin T Choo KKR Liu CZ He D. Blockchain in healthcare applications: research challenges and opportunities. J Netw Comput Appl. (2019) 135:62–75. 10.1016/j.jnca.2019.02.02730914045

[87] Azaria A Ekblaw A Vieira T Lippman A. MedRec: using blockchain for medical data access and permission management. In: 2016 2nd International Conference on Open and Big Data (OBD). Vienna (2016). p. 25–30.

[88] Nebula Genomics. Available online at: https://www.nebula.org/ (accessed November 12, 2018).

[89] O'Leary DE. Artifi cial intelligence and big data what is big data? IEEE Intell Syst. (2013) 28:66–9. 10.1109/MIS.2013.39

[90] O'Neil C. Weapons of Math Destruction: How Big Data Increases Inequality and Threatens Democracy. Philadelphia: Crown Publishing Group (2016).

[91] Castelvecchi D. The black box 2 0 |. Nature. (2016) 538:20–3. 10.1038/538020a 27708329

[92] Goodfellow IJ Shlens J Szegedy C. Explaining and harnessing adversarial examples. In: 3rd Int International Conference on Learning Representations 2015. San Diego, CA (2015). p. 1–11.

[93] Barredo Arrieta A Díaz-Rodríguez N Del Ser J Bennetot A Tabik S Barbado A. Explainable explainable artificial intelligence (XAI): concepts, taxonomies, opportunities and challenges toward responsible AI. Inf Fusion. (2020) 58:82–115. 10.1016/j.inffus.2019.12.012

[94] Thorogood A Simkevitz H Phillips M Dove ES Joly Y. Protecting the privacy of Canadians' health information in the cloud. Can J Law Technol. (2013) 14:8.

[95] PIPEDA in Brief – Office of the Privacy Commissioner of Canada. Available online at: https://www.priv.gc.ca/en/privacy-topics/privacy-laws-in-canada/the-personal-information-protection-and-electronic-documents-act-pipeda/pipeda_brief/ (accessed November 15, 2018).

[96] Summary of Privacy Laws in Canada. Office of the Privacy Commissioner of Canada. Available online at: https://www.priv.gc.ca/en/privacy-topics/privacy-laws-in-canada/02_05_d_15/#heading-0-0-3-1 (accessed January 23, 2020).

[97] HIPAA Compliance Checklist. Available online at: https://www.hipaajournal.com/hipaa-compliance-checklist/ (accessed January 22, 2020).

[98] GDPR. Art. 4 GDPR - Definitions - GDPR.eu. (2021). Available online at: https://gdpr.eu/article-4-definitions/?cn-reloaded=1 (accessed January 20, 2021).

[99] Velmovitsky PE Miranda PADSES Vaillancourt H Donovska T Teague J Morita PP. Blockchain and IoT: a conceptual framework for a blockchain consent platform in active assisted living. J Med Internet Res. (2020) 22:e20832. 10.2196/20832 33275111 PMC7748951

[100] Cavoukian A. Frequently Asked Questions: Personal Health Information Protection Act. (2004). p. 1–42. Available online at: https://www.ipc.on.ca/images/Resources/hfaq-e.pdf%5Cnhttp://www.ipc.on.ca/images/resources/hguide-e.pdf

[101] Emam El K Rodgers S Malin B. Anonymising and sharing individual patient data. BMJ. (2015) 350:h1139. 10.1136/bmj.h1139 25794882 PMC4707567

[102] Iron K Sykora K. Health Services Data, Sources and Examples. The Institute for Clinical Evaluative Sciences Data Repository (2019). p. 47–59.

[103] Sedayao J Bhardwaj R Gorade N. Making big data, privacy, and anonymization work together in the enterprise: experiences and issues. In: 2014 IEEE International Congress on Big Data. Anchorage, AK (2014). p. 601–7.

[104] Chamberlayne R Green B Barer ML Hertzman C Lawrence WJ Sheps SB. Creating a population-based linked health database: a new resource for health services research. Can J Public Heal. (1998) 89:270–3. 10.1007/BF03403934 9735524 PMC6990342

[105] Compilor. Pseudonymization and Anonymization of Personal Data – What is the Difference? | Complior. Available online at: https://complior.se/pseudonymization-and-anonymization-of-personal-data-what-is-the-difference/ (accessed January 29, 2020).

[106] Association CM. Data Privacy - What the Canadian Consumer Really Thinks (2018).

[107] Jagsi R Griffith KA Jones RD Krenz C Gornick M Spence R. Effect of public deliberation on patient attitudes regarding consent and data use in a learning health care system for oncology. J Clin Oncol. (2019) 37:3203–11. 10.1200/JCO.19.01693 31577472 PMC6881101

[108] Chowkwanyun M Bayer R Galea S. “Precision” public health — between novelty and hype. N Engl J Med. (2018) 379:1398–400. 10.1056/NEJMp1806634 30184442

[109] Horton R. Offline: in defence of precision public health. Lancet. (2018) 392:1504. 10.1016/S0140-6736(18)32741-7 30496048

[110] Chowkwanyun M Bayer R Galea S. Precision public health: pitfalls and promises. Lancet. (2019) 393:1801. 10.1016/S0140-6736(18)33187-8 31057162

